# CRITICAL ASPECTS OF MOBILITY FOR IMPROVING PATIENT OUTCOMES AFTER CARDIAC SURGERY

**DOI:** 10.1093/geroni/igz038.1759

**Published:** 2019-11-08

**Authors:** Tiffany Tsay, Jennifer A Schrack, Allyse Depenbrock, Amal A Wanigatunga, Jacek Urbanek, Charles H Brown

**Affiliations:** 1 Johns Hopkins Bloomberg School of Public Health, Baltimore, Maryland, United States; 2 Johns Hopkins University, Baltimore, Maryland, United States; 3 John Hopkins University School of Medicine, Department of Medicine, Division of Geriatric Medicine and Gerontology, Center on Aging and Health, Baltimore, Maryland, United States; 4 Johns Hopkins University School of Medicine, Baltimore, Maryland, United States

## Abstract

Physical activity after major cardiac surgery has been associated with length of stay, discharge location, risk of readmission, and functional change. However, in-hospital mobility is not currently assessed in a standardized way with nurse reports being the primary mechanism of tracking patient activity. Furthermore, it is unclear whether it is the total amount, frequency, or type of activity that is most important for improving patient outcomes post-surgery. To better understand the duration, frequency, and intensity of patient activity post-cardiac surgery, we conducted an observational study of 206 patients using a wrist-worn accelerometer and ankle-worn pedometer. Patients with lower levels of average daily pedometer-based ambulation in the first four days post-surgery, when compared to counterparts who ambulated more, had higher odds of a longer length of stay (OR=4.55, p<0.0001) or being discharged to rehab vs. home (OR=7.7 p=0.012), independent of age, race, bypass time, and EuroSCORE (cardiac surgery risk score). Engaging in an average of less than two bouts of accelerometer-derived activity lasting 5 minutes or more each day was associated with higher odds of having a longer length of stay (OR=2.69, p=0.008) or being discharged to rehab vs. home (OR=20.9, p=0.019). A slower speed of recovery during the first four postoperative days, characterized by a smaller increase in pedometer-based ambulation with each successive day, was also associated with higher odds of being discharged to rehab vs. home (OR=6.62, p=0.008). Further research is needed to develop appropriate frequency and activity thresholds for use as intervention tools to improve patient outcomes post-surgery.

